# Racial Disparities in Associations between Neighborhood Demographic Polarization and Birth Weight

**DOI:** 10.3390/ijerph17093076

**Published:** 2020-04-28

**Authors:** Kelvin C. Fong, Maayan Yitshak-Sade, Kevin J. Lane, M. Patricia Fabian, Itai Kloog, Joel D. Schwartz, Brent A. Coull, Petros Koutrakis, Jaime E. Hart, Francine Laden, Antonella Zanobetti

**Affiliations:** 1 Department of Environmental Health, Harvard TH Chan School of Public Health, Boston, MA 02215, USA; myitshak@hsph.harvard.edu (M.Y.-S.); joel@hsph.harvard.edu (J.D.S.); bcoull@hsph.harvard.edu (B.A.C.); petros@hsph.harvard.edu (P.K.); rejch@channing.harvard.edu (J.E.H.); francine.laden@channing.harvard.edu (F.L.); azanobet@hsph.harvard.edu (A.Z.); 2 School of Forestry and Environmental Studies, Yale University, New Haven, CT 06511, USA; 3 Department of Environmental Health, School of Public Health, Boston University, Boston, MA 02218, USA; klane@bu.edu (K.J.L.); pfabian@bu.edu (M.P.F.); 4 Department of Geography and Environmental Development, Ben-Gurion University of the Negev, 8410501 Beer Sheva, Israel; ikloog@bgu.ac.il; 5 Department of Epidemiology, Harvard TH Chan School of Public Health, Boston, MA 02215, USA; 6 Department of Biostatistics, Harvard TH Chan School of Public Health, Boston, MA 02215, USA; 7 Department of Medicine, Channing Division of Network Medicine, Brigham and Women’s Hospital and Harvard Medical School, Boston, MA 02215, USA

**Keywords:** neighborhood, birth weight, disparities, racial/ethnic, privilege, social environment, social stress, effect modification

## Abstract

Neighborhood demographic polarization, or the extent to which a privileged population group outnumbers a deprived group, can affect health by influencing social dynamics. While using birth records from 2001 to 2013 in Massachusetts (*n* = 629,675), we estimated the effect of two demographic indices, racial residential polarization (RRP) and economic residential polarization (ERP), on birth weight outcomes, which are established predictors of the newborn’s future morbidity and mortality risk. Higher RRP and ERP was each associated with higher continuous birth weight and lower odds for low birth weight and small for gestational age, with evidence for effect modification by maternal race. On average, per interquartile range increase in RRP, the birth weight was 10.0 g (95% confidence interval: 8.0, 12.0) higher among babies born to white mothers versus 6.9 g (95% CI: 4.8, 9.0) higher among those born to black mothers. For ERP, it was 18.6 g (95% CI: 15.7, 21.5) higher among those that were born to white mothers versus 1.8 g (95% CI: −4.2, 7.8) higher among those born to black mothers. Racial and economic polarization towards more privileged groups was associated with healthier birth weight outcomes, with greater estimated effects in babies that were born to white mothers than those born to black mothers.

## 1. Introduction

The demographic polarization of a neighborhood describes the extent to which a population group outnumbers another [1,2]. Population groups at opposite ends of privilege are often counted to determine demographic polarization; thus, it is related to a neighborhood’s social dynamics, which, in turn, impacts residents’ health [3,4]. In a given geographic area, the ratio of residents belonging to a privileged group as compared to a deprived group serves as an index of demographic polarization. For the United States (US), race and income serve as demarcations for calculating demographic polarization in a given neighborhood (i.e., Census tract). Specifically, racially white residents are considered to be a privileged group, while black residents are considered as a deprived group; residents whose household income is in the top quartile are considered to be privileged, while those whose household income is in the lowest quartile are considered deprived. The extent to which a neighborhood is polarized towards a privileged or deprived population group is linked to the access and amount of resources, which are consequential to health, in addition to social stress among its residents. 

To date, few studies have investigated the association between neighborhood demographic polarization and health. Of those that are published, demographic polarization towards more privileged groups was associated with better health outcomes, such as decreased risk for hypertension [5], preterm birth [3], and premature mortality [6]. In this study, we investigate the association between neighborhood racial/economic polarization and birth weight outcomes: continuous birth weight, low birth weight (<2500 g; LBW), and small for gestational age (<10th percentile of birth weight for the newborn’s sex and gestational age; SGA). Birth weight is a ubiquitous measurement and it is predictive of health of the newborn; babies of lower birth weight have a higher risk for chronic diseases, developmental disorders, and premature mortality [7,8,9,10]. Neighborhood demographic polarization is related to access to health-promoting resources, such as health education or services [11]. For mothers living in more deprived neighborhoods during pregnancy, fetal growth might not be optimal, leading to lower birth weight and increased odds of LBW or SGA. Thus, we hypothesize that babies born to mothers who resided in neighborhoods polarized to more privileged groups would have higher birth weights and lower odds for LBW/SGA, thus indicating lower future morbidity and mortality risks. Although prior studies have shown that neighborhood composition, such as higher unemployment percentage or poverty, are associated with lower birth weight [12], such measures do not capture the extremes of privilege and deprivation simultaneously [11]. Demographic polarization fills this gap by quantifying the extent to which those belonging to a privileged group outnumber those belonging to a deprived group, and vice versa. Furthermore, demographic polarization might better describe the social dynamics in a neighborhood, which influences the amount of social stress experienced by expectant mothers, impacting fetal growth. Past studies have found that maternal race is associated with birth weight outcomes: those born to black mothers are more likely to have lower birth weight and to be LBW or SGA when compared to those born to white mothers [12,13,14]. As different demographic groups (e.g., race) were likely to experience very different levels of social stress and challenges related to demographic polarization, we tested to see whether the estimated effect of demographic polarization on birth weight outcomes was modified by maternal race.

## 2. Materials and Methods

### 2.1. Neighborhood Demographic Polarization

We calculated demographic polarization based on race using five-year estimates from the 2010 American Community Survey, which we termed racial residential polarization (RRP), and demographic polarization based on income, which we termed economic residential polarization (ERP) [15]. In each Census tract, we calculated RRP, as in Equation (1) [1,2]:

RRP_i_ = (W_i_ − B_i_)/T_i_ (1)

where W_i_ represents the number of non-Hispanic white residents, B_i_ the number of non-Hispanic black residents, and T_i_ the total number of residents in Census tract i. In other words, it represents the difference between the number of residents that belong to the privileged group and the number belonging to the deprived group, divided by the total number of residents in a given Census tract. For ERP, the calculation is similar: W_i_ is replaced with the number of households earning ≥ $100,000 per year, B_i_ with the number of households earning < $25,000 per year, and T_i_ with the total number of households in Census tract i. These two annual income cut points approximately correspond to the 75th and 25th percentiles of annual household income in Massachusetts in 2010 [15]. RRP and ERP each have a theoretical range from −1 (completely polarized towards the deprived group) to 1 (completely polarized towards the privileged group). For a given Census tract, −1 indicates that 100 percent of the population or households was concentrated into the deprived group, while 1 indicates that 100 percent of the population or households was concentrated into the privileged group; 0 indicates that there was a 1 to 1 ratio, or equal numbers in the privileged group and the deprived group. In Massachusetts, RRP was moderately correlated with ERP (Table S1). As expected, RRP was more strongly correlated with racial compositional measures, while ERP was correlated with household income compositional measures. 

### 2.2. Study Population

We obtained records from the Massachusetts Department of Public Health for all births between 1 January 2001 and 31 December 2013 (*n* = 978,225). We excluded those with missing address information (*n* = 23,943), with a birth weight below 500 g (*n* = 772), not live births (*n* = 8621), not singletons (*n* = 42,186), and not full-term (gestational age not between 37 and 44 weeks; *n* = 77,036). We excluded a further 154,296 births (19 percent) born to mothers of other racial/ethnic groups, as we were interested in comparing birth outcomes in those born to non-Hispanic white and non-Hispanic black mothers. In addition to birth weight, the birth records included information about the pregnancy itself, such as gestational age, as well as maternal characteristics, such as race/ethnicity, smoking, medical conditions before or during pregnancy, and highest educational level attained. After excluding those with missing covariate data (*n* = 41,696), the final sample size was 629,675. The Massachusetts Department of Public Health geocoded each maternal residential address against TomTom Multinet using AccuMail address and Zip code. These geocodes allowed us to link each birth to its residential Census tract. The Massachusetts Department of Public Health approved the use of these data.

### 2.3. Statistical Analysis

We constructed separate regression models to determine the association between RRP or ERP and three birth weight outcomes: continuous birth weight, low birth weight (<2500 g; LBW), and small for gestational age (<10th percentile of birth weight for the newborn’s sex and gestational age; SGA). For birth weight, we built linear mixed models; we built logistic mixed models for LBW and SGA. These mixed models included a random intercept for Census tract to account for possible geographical clustering and confounding by unmeasured spatial variables.

We started with univariate analyses quantifying the unadjusted association between RRP or ERP and each birth weight outcome. Subsequently, we built multivariate models adjusted for covariates selected *a priori* based on those used in prior birth weight studies [16,17,18]. These included maternal age, maternal race/ethnicity, maternal marital status, maternal smoking prior to or during pregnancy, maternal education, parity, maternal diabetes, gestational diabetes, maternal chronic high blood pressure, maternal high blood pressure during pregnancy, Kessner index of adequacy of prenatal care [19], birth mode of delivery, clinical gestational age, year of the birth, newborn sex, and Medicaid status. In addition, we adjusted for particulate air pollution less than or equal to 2.5 µm in aerodynamic diameter (PM_2.5_), since maternal exposure to PM_2.5_ during pregnancy is negatively associated with birth weight [20,21]. Each birth’s PM_2.5_ exposure during the entire pregnancy was estimated by calculating the average of daily PM_2.5_ predictions while using the maternal residential address and gestational age. The PM_2.5_ data came from a hybrid prediction model that combined land-use variables and remote sensing measurements from satellites to estimate PM_2.5_ daily at a 1 km × 1 km resolution. It was calibrated with PM_2.5_ monitoring measurements and it was performed with high accuracy with temporal, spatial, and combined out-of-sample R^2^ consistently above 0.8 [22]. In the models with RRP as the explanatory variable of interest, we additionally adjusted for Census tract median household income; in the models with ERP as the explanatory variable of interest, we additionally adjusted for Census tract percentage non-Hispanic black population. As sensitivity analyses, we included both Census tract median household income and percentage non-Hispanic black population in RRP and ERP models. We conducted supplementary analyses where a covariate is omitted from the full model one at a time, and then recorded the estimated effect of RRP or ERP, to assess each covariate’s contribution to confounding the relationship between either RRP or ERP and birth weight.

We evaluated disparities in the estimated effects of RRP or ERP on birth weight outcomes among those born to white mothers when compared to black mothers by including an interaction term between individual maternal race and RRP or ERP in fully-adjusted models. We then computed the point estimate of the explanatory variable (i.e., RRP and ERP) along with its 95 percent confidence interval (95% CI). We were concerned about the modifiable areal unit problem [23], so we conducted sensitivity analyses with RRP and ERP being calculated at the Census block group level, which is a subdivision of a smaller geographical scale when compared to Census tract. We conducted all data linkage and statistical analyses while using R 3.5.3 [24]. All of the calculated *P* values were two-sided.

## 3. Results

We analyzed a total of 629,675 births, of which 89.5 percent were born to white mothers and the remainder to black mothers (Table 1). RRP had a median value of 0.88 with an interquartile range (IQR) of 0.24 and the median ERP was 0.12 with an IQR of 0.37; therefore, there was more variation in demographic polarization by income than by race. No Massachusetts Census tract had an undefined RRP or ERP, since each had a non-zero number of either group. More than two-thirds of the births were to mothers who were married and more than one-third were eligible for Medicaid. Over 80 percent of the newborns received adequate prenatal care according to the Kessner index and over 90 percent of the mothers had at least a high school education.

Higher RRP or ERP was each associated with increased continuous birth weight and lower odds for LBW and SGA (Table 2). The magnitude of the estimated effect of either RRP or ERP was much larger in unadjusted univariate regression models than the fully adjusted mixed model, suggesting that the covariates included in the final model *a priori* were confounders. In supplementary analyses, we found that maternal smoking, maternal education, clinical gestational age, Medicaid eligibility, parity, and PM_2.5_ to be of the most substantial sources of confounding (Figure S1).

**Figure 1:**
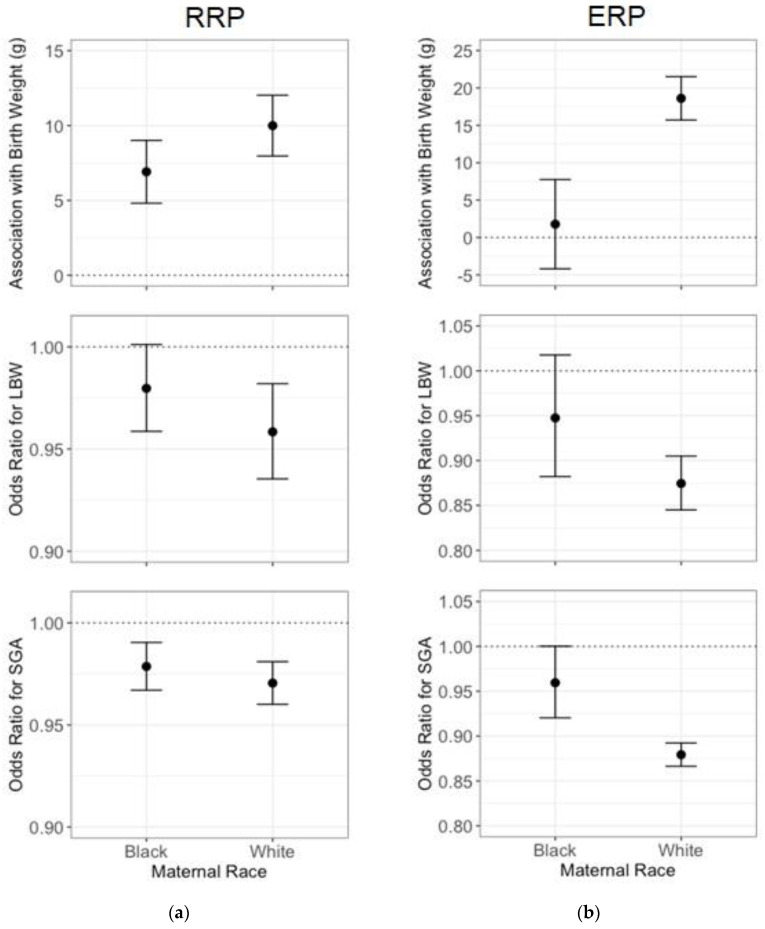
Association between an Interquartile Range (IQR) Difference in Census Tract (**a**) Racial Residential Polarization (RRP; IQR = 0.24) or (**b**) Economic Residential Polarization (ERP; IQR = 0.37) and Continuous Birth Weight, Odds for Low Birth Weight (LBW), and Odds for Small for Gestational Age (SGA) in Massachusetts from 2001 to 2013 (*n* = 629,675). Estimated effects and 95% confidence intervals are presented separately for those born to black mothers and those born to white mothers. Model covariates include: particulate matter under 2.5 µm in aerodynamic diameter (PM_2.5_), clinical gestational age, mother’s age, infant sex, year of birth, maternal marital status, Medicaid status, maternal smoking, gestational diabetes, other diabetes, high blood pressure during pregnancy, chronic high blood pressure, parity, mode of delivery, Kessner index for adequacy of prenatal care, and maternal education.

We observed effect modification by maternal race. There was strong evidence for an interaction between RRP and maternal race for continuous birth weight (*p* = 0.0004); Figure 1a shows that an IQR increase in RRP (0.24) was associated with 6.9 g (95% CI: 4.8, 9.0) higher birth weight among those born to black mothers as compared to 10.0 g (95% CI: 8.0, 12.0) higher birth weight among those born to white mothers. For odds ratios for LBW per IQR increase in RRP (*p* for interaction = 0.17), the black-white disparity was 0.98 (95% CI: 0.96, 1.00) as compared to 0.96 (95% CI: 0.94, 0.98); for SGA (*p* for interaction = 0.29), it was 0.98 (95% CI: 0.97, 0.99) compared to 0.97 (95% CI: 0.96, 0.98). There was also strong evidence for an interaction between ERP and maternal race for continuous birth weight (*p* < 0.0001); Figure 1b presents the associations between an IQR increase in ERP and birth weight outcomes, stratified by maternal race. The black-white disparity in the estimated effects were as follows: 1.8 g (95% CI: −4.2, 7.8) among those born to black mothers when compared to 18.6 g (95% CI: 15.7, 21.5) among those born to white mothers, OR for LBW 0.95 (95% CI: 0.88, 1.02) compared to 0.87 (95% CI: 0.84, 0.90) with a *P* for interaction of 0.0274, and OR for SGA 0.96 (95% CI: 0.92, 1.00) as compared to 0.88 (95% CI: 0.87, 0.89) with a *p* for interaction < 0.0001. The observed associations were similar in sensitivity analyses while using the RRP or ERP measures at the Census block group geographical scale (Figures S2 and S3). Sensitivity analyses that included both Census tract median household income and percentage non-Hispanic black population in RRP and ERP models found similar estimated effects of RRP and ERP that were slightly larger in magnitude. However, they did not show as strong evidence for effect modification by maternal race, with a larger *P* for interaction in birth weight, LBW, and SGA models. For this reason and concern for over-adjustment for similar measures, the final RRP model did not include percentage non-Hispanic black population, and the final ERP model did not include median household income.

## 4. Discussion

Our analysis of full-term, live singleton births in Massachusetts from 2001 to 2013 found that demographic polarization towards more privileged groups, as indicated by higher RRP and ERP values, was positively associated with continuous birth weight and negatively associated with odds of LBW and SGA. In fully adjusted models, the associations between either RRP or ERP and odds for LBW and SGA were similar. Importantly, the magnitudes of the estimated effects on all outcomes were larger among those born to white mothers as compared to those born to black mothers, demonstrating a black-white disparity in the estimated health effects of racial and income demographic polarization.

Demographic polarization towards more privileged racial or income groups was associated with better health, as indicated by higher birth weight and lower odds for LBW and SGA. This was likely related to the correlation between higher RRP/ERP values and neighborhood socioeconomic status (SES). Expectant mothers living in Census tracts with higher RRP/ERP values were likely to have better access to healthcare and other health-promoting activities [2]. These factors contribute to a healthy uterine environment and they protect against birth weight detriments [25]. Although the exact mechanisms are not clear, it is postulated that residents of areas with strongly-negative RRP/ERP values, or polarization towards deprived groups, experience chronic psychological stress from neighborhood exposures, such as higher rates of violent crime [26]. Social stress leads to higher anxiety, decreased metabolic function, and lower general health, all of which are linked to a risk for birth weight detriments [26,27]. As part of the physiological response to stress, expectant mothers experience higher levels of catecholamines, which can lead to placental hypoperfusion, resulting in lower oxygen and nutrient delivery to the fetus [28]. Our findings are consistent with prior studies on demographic polarization and adverse birth outcomes; these studies found the highest rates of preterm birth and infant mortality in those born to mothers residing in areas with most negative RRP and ERP in New York City and California [3,29,30]. Although we are not aware of an existing study investigating RRP or ERP and our birth weight outcomes, numerous studies have found that higher neighborhood SES is associated with higher birth weights and lower odds for LBW and SGA [18,28,31]. More broadly, higher RRP or ERP have each been associated with health benefits, such as decreased hypertension incidence and risk for premature mortality [5,6]. In summary, demographic polarization towards more privileged groups, as indicated by higher RRP and ERP, was associated with healthier birth weight outcomes.

Our results demonstrate disparity by maternal race in estimated effects of RRP and ERP on birth weight outcomes. The magnitudes of the association between RRP or ERP on continuous birth weight, odds for LBW and SGA were smaller among those born to black mothers than those born to white mothers. This black-white health disparity could stem from differences in social interactions that black mothers experience when compared to their white counterparts. Specifically, black mothers were likely to have experienced higher amounts of negative social interactions than white mothers [3]. The negative health effects from structural racism manifest themselves in differential access to services, goods, and opportunities by race. In US counties with higher racial prejudice, LBW among those born to black mothers was 14 percent higher than those born to white mothers [32]. Prior studies also found other measures of demographic polarization to be associated with adverse birth outcomes, such as preterm birth and infant mortality, and that the outcomes were worse among those born to black mothers when compared to white mothers [33]. Although they did not use the same measures of demographic polarization, prior studies found that mothers who resided in low-income communities were more likely to give birth to LBW babies than those who resided in higher-income communities, with higher black-white disparities among low-income community residents [34]. More generally, prior studies have found that those born to black mothers, as compared to those born to white mothers, have a higher risk for adverse birth outcomes and lower birth weight [13,32]. Our findings suggest that, although individual maternal characteristics, such as highest education attained, contribute to black-white disparities in birth weight [21,32], demographic polarization in the maternal residential neighborhood also influences birth weight outcomes. When comparing our results for RRP versus ERP, we found that the black-white disparity was larger with ERP than with RRP. This contrasts prior work that found racial polarization to be a stronger predictor of health inequities than economic polarization [3]. This inconsistency with prior findings could be due to different methods and effect modification by geographic region; prior studies on the associations between demographic polarization and birth outcomes occurred in New York City and California [3,29]. Effect modification by region is likely, since social context, health infrastructure, and health policy differ by geographic area. To sum up, our study found evidence of effect modification by maternal race in the relationship between demographic polarization and birth weight outcomes. This finding is consistent with prior literature and is expected, since a member of a deprived group is likely to face different challenges when compared to a member of a privileged group, even if both were residing in the same area.

This study had several strengths and limitations. First, our choice to calculate RRP and ERP at the Census tract level could have led to biased estimates due to the modifiable area unit problem since geographic boundaries are arbitrary [23]. However, we conducted sensitivity analyses using RRP and ERP calculated at the Census block group level and found very similar results (Figures S2 and S3), suggesting that the modifiable area unit problem was a minimal source of bias. Secondly, although we had a large sample size covering over a decade in Massachusetts, our results have limited generalizability to births in other parts of the US and the world. Nonetheless, the direction of associations is likely to be consistent, but the magnitudes of associations between RRP or ERP and birth weight outcomes could vary across the US due to differences in demographic makeup, social dynamics, and health policies [3]. Another note regarding generalizability is that, since we restricted our study population to full-term live singletons, our conclusions regarding associations between RRP or ERP and birth outcomes, as well as the black-white disparities, are limited to those related to term birth weight, and not to adverse birth outcomes, such as preterm birth and infant mortality, which have previously been explored [3,29]. The mechanisms through which demographic polarization affect preterm birth, infant mortality, and birth weight could be related, but also potentially distinct. A separate limitation was potential exposure misclassification from using the 2006–2010 American Community Survey to calculate RRP and ERP for births from 2001 to 2013. We expect the potential bias to be non-differential, given that 2006–2010 was around the midpoint of the study period and that the five-year estimates are the most stable American Community Survey data [15]. Exposure misclassification was also possible due to missing or misreported maternal residential addresses, but it was unlikely that reporting errors were associated with either RRP or ERP and birth weight. Another potential shortfall of using reported maternal residential addresses to determine demographic polarization is that residential attainment in the US is not random and there are factors, some unaccounted for in the present study, which influence where one lives. More generally, although we were able to adjust for many individual characteristics related to each birth, the results were prone to residual confounding. For example, we were not able to adjust for variables, such as maternal or family income due to data unavailability. We expect the bias stemming from residual confounding to be non-differential and not affecting the direction of observed associations between demographic polarization and birth weight outcomes. On the other hand, while prior studies on demographic polarization and birth outcomes did not adjust for confounding from environmental exposures, our study distinguishes itself by accounting for PM_2.5_, an exposure that has been linked to birth weight detriments [21]. Moreover, to our knowledge, this study is the first investigate demographic polarization in relation to birth weight outcomes. Demographic polarization indices, such as RRP and ERP, are distinguished from traditional compositional measures, because they simultaneously capture extremes of privilege and deprivation, and they may additionally indicate social stress encountered by neighborhood residents. Although RRP was strongly correlated with racial compositional measures, and ERP with economic compositional measures (Table S1), this might not be the case in states where there is a stronger imbalance between members that belong to a privileged group when compared to a deprived group, or where there are higher populations of neither the privileged nor the deprived groups. We expect that the direction of association between demographic polarization towards more privileged groups and birth weight outcomes to be the same Massachusetts and other states, but we expect the magnitudes of these associations to differ. A study of demographic polarization and birth weight outcomes in other parts of the U.S. is worthy of future research. Further analyses should also consider adjusting for exposures, such as access to green spaces, which has been linked to birth weight outcomes [35], to reduce residual confounding and elucidate the interplay between neighborhood demographic polarization, environmental exposures, and health, as well as how they relate to health disparities by race.

## 5. Conclusions

Within full-term, live singleton births in Massachusetts from 2001 to 2013, Census tract racial and economic demographic polarization towards more privileged groups was associated with a higher continuous birth weight and lower odds for low birth weight and small for gestational age. The estimated effects were smaller among those born to black mothers than those born to white mothers, demonstrating a health disparity by race.

## Figures and Tables

**Table 1:** Characteristics of Full-Term Live Singleton Births born to White and Black Mothers in Massachusetts from 2001 to 2013.

Variable	Overall
Total Births (*n*)	629,675
**Continuous Variables (median ± interquartile range)**	
Birthweight in Grams	3459 ± 623
Average PM_2.5_ over entire Pregnancy (µg/m^3^)	10.2 ± 2.3
Clinical Gestational Age in Weeks	39 ± 1
Maternal Age in Years	31 ± 8.2
Racial Residential Polarization (RRP)	
at Census Block Group	0.88 ± 0.27
at Census Tract	0.86 ± 0.24
Economic Residential Polarization (ERP)	
at Census Block Group	0.12 ± 0.43
at Census Tract	0.12 ± 0.37
**Binary and Categorical Variables (%)**	
Infant Sex = Female	49.1
Maternal Marital Status = Married	72.0
Medicaid Status = Yes	28.2
Smoking During or Prior to Pregnancy	15.2
Gestational Diabetes	3.7
Other Diabetes	0.8
High Blood Pressure during Pregnancy	3.6
Chronic High Blood Pressure	1.3
Parity: First-Pregnancy	45.2
Mode of Delivery	
Vaginal	64.9
Forceps	0.6
Vacuum	3.5
First Caesarian Birth	17.1
Repeat Caesarian	12.4
Vaginal Birth after Previous Caesarean Birth	1.5
Maternal Race	
White	89.5
Black	10.5
Kessner Index for Adequacy of Prenatal Care	
Adequate	80.3
Intermediate	15.6
Inadequate	3.0
No Prenatal Care	1.2
Maternal Education	
Less than High School	7.4
High School	22.8
Some College	23.7
College	28.5
Advanced Degree	17.6

**Table 2:** Associations between an Interquartile Range Increase in Census Tract Racial Residential Polarization (RRP) or Economic Residential Polarization (ERP) and Birth Weight Outcomes.

		Outcome		
-	-	Birth Weight (g)	LBW (OR) ^1^	SGA (OR)
Unadjusted	RRP ^2^	134.3 (130.6, 137.9) ^3^	0.86 (0.85, 0.87)	0.94 (0.93, 0.94)
	ERP	175.1 (170.8, 179.3)	0.68 (0.67, 0.70)	0.76 (0.75, 0.77)
Full Model ^4^	RRP	8.7 (7.4, 10.0)	0.96 (0.94, 0.98)	0.97 (0.96, 0.98)
	ERP	17.0 (14.1, 19.8)	0.88 (0.86, 0.91)	0.88 (0.87, 0.89)

^1^ Abbreviations: LBW, low birth weight; SGA, small for gestational age; OR, odds ratio. ^2^ The interquartile range for RRP was 0.24 and the IQR for ERP was 0.43. ^3^ Point estimates and 95% confidence intervals are shown. For birth weight, the estimated effect is in grams; for low birth weight and small for gestational age, the odds ratio is given. ^4^ The full model adjusted for particulate matter less than or equal to 2.5 µm in aerodynamic diameter (PM_2.5_), clinical gestational age, mother’s age, infant sex, year of birth, maternal marital status, Medicaid status, maternal smoking, gestational diabetes, other diabetes, high blood pressure during pregnancy, chronic high blood pressure, parity, mode of delivery, Kessner index for adequacy of prenatal care, and maternal education.

## Supplementary Materials

The following are available online at https://www.mdpi.com/1660-4601/17/9/3076/s1, Table S1: Pearson Correlations between Neighborhood Demographic Polarization Indices and Composition. Figure S1: Assessment of Confounding by Covariates in the Relationship between Census Tract Racial Residential Polarization or Economic Residential Polarization and Birth Weight in Massachusetts from 2001 to 2013, Figure S2: Association between an Interquartile Range Increase in Census Block Group Racial Residential Polarization and Continuous Birth Weight, Odds for Low Birth Weight, and Odds for Small for Gestational Age in Massachusetts from 2001 to 2013, Figure S3: Association between an Interquartile Range Increase in Census Block Group Economic Residential Polarization and Continuous Birth Weight, Odds for Low Birth Weight, and Odds for Small for Gestational Age in Massachusetts from 2001 to 2013.
