# Supplementary material for: Racial Disparities in Associations between Neighborhood Demographic Polarization and Birth Weight
Source: Int J Environ Res Public Health. 2020 Apr 28;17(9):3076. doi: 10.3390/ijerph17093076 (PMC7246784; doi:10.3390/ijerph17093076)

Table S1.

Pearson Correlations between Neighborhood Demographic Polarization Indices and Composition. Correlations with Census-tract level racial residential polarization (RRP) and economic residential polarization (ERP) are given.

	**RRP**	**ERP**
ERP	0.50	n/a
% black population	-0.92	-0.39
% white population	0.97	0.53
% high income ($100,000 per year) households	0.43	0.95

Figure S1.
Assessment of Confounding by Covariates in the Relationship between Census Tract Racial Residential Polarization (RRP) or Economic Residential Polarization (RRP) and Birth Weight in Massachusetts from 2001 to 2013 (n = 629,675). Estimated associations between an IQR increase in RRP (0.27; A) or RRP (0.43; B) and changes in continuous birth weight along with the 95% confidence intervals are shown. The estimate for the full model is shown in red and the estimates for models omitting the covariate are in black.

A)

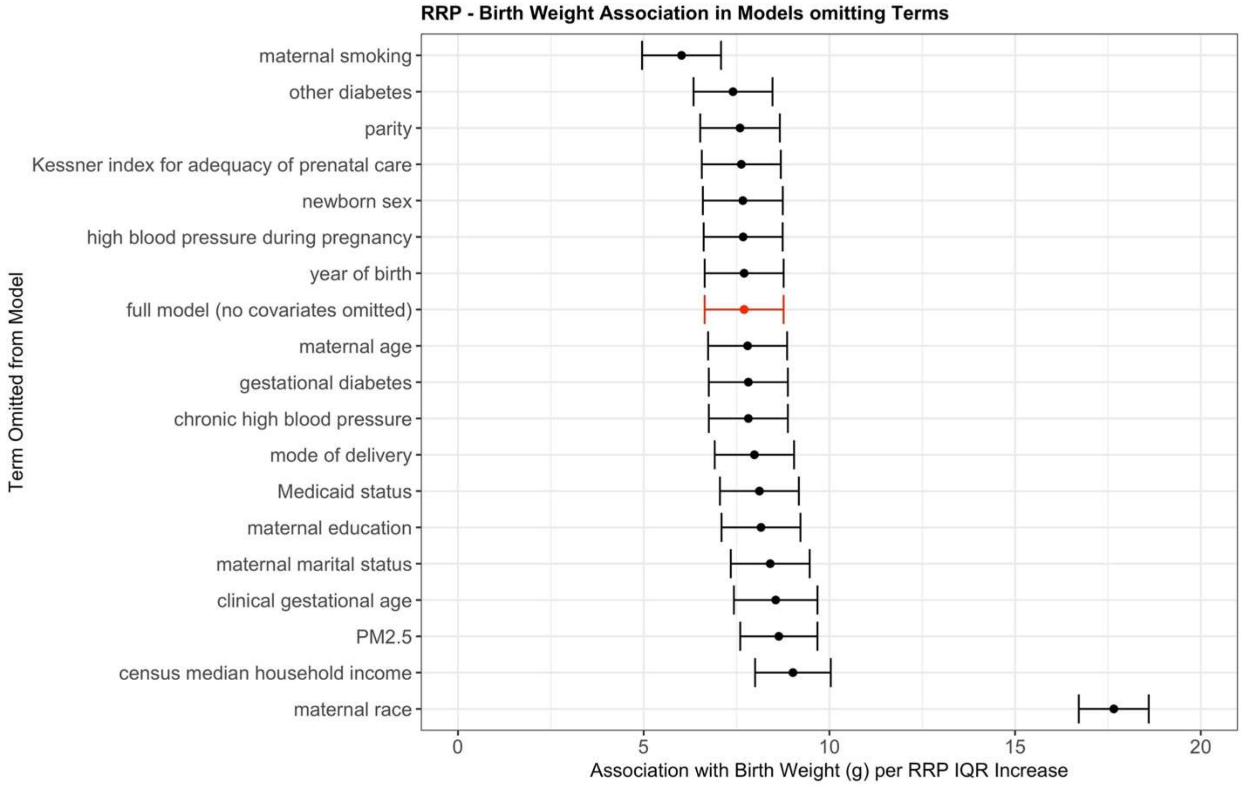

B)

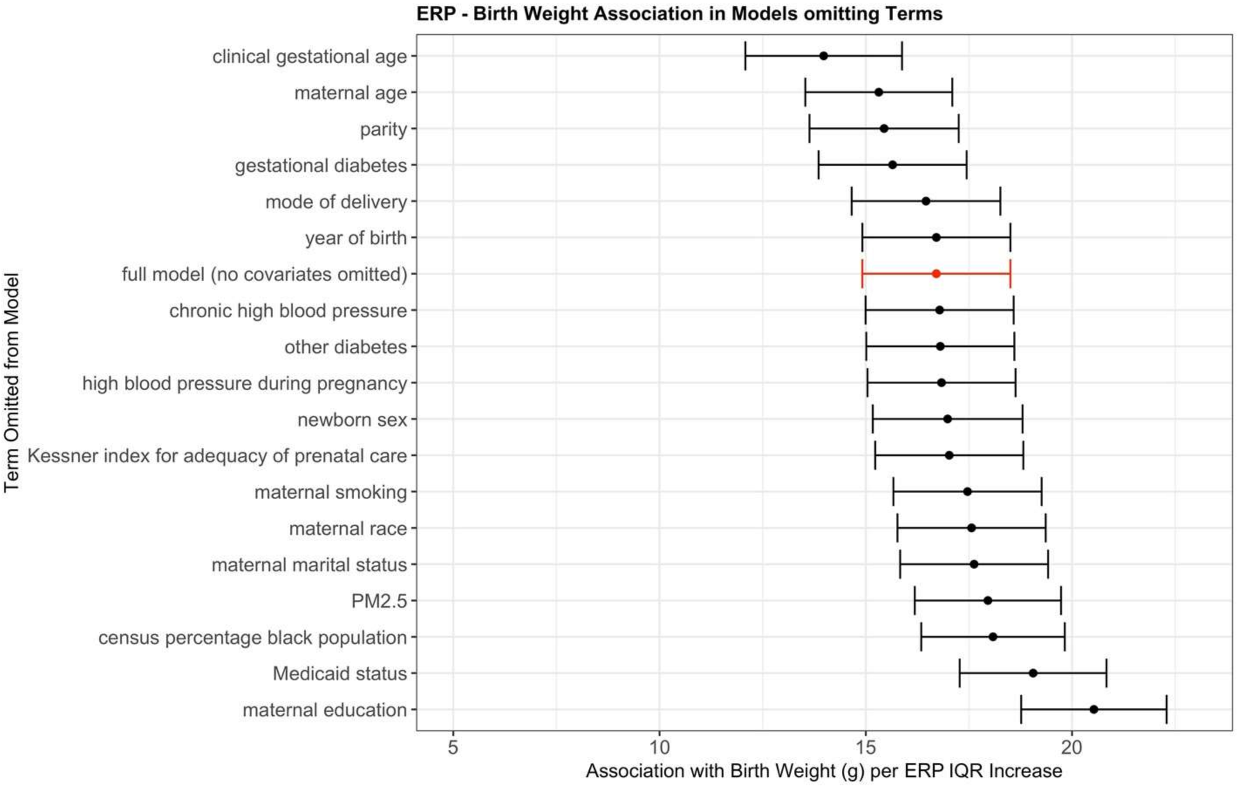
ERP - Birth Weight Association in Models omitting Terms
clinical gestational age
maternal age
parity
gestational diabetes
mode of delivery
year of birth
full model (no covariates omitted)
chronic high blood pressure
other diabetes
high blood pressure during pregnancy
newborn sex
Kessner index for adequacy of prenatal care
maternal smoking
maternal race
maternal marital status
PM2.5
census percentage black population
Medicaid status
maternal education
Term Omitted from Model
5
10
15
20
Association with Birth Weight (g) per ERP IQR Increase

Figure S2.
Association between an Interquartile Range Increase in Census Block Group Racial Residential Polarization (RRP; 0.27) and Continuous Birth Weight, Odds for Low Birth Weight (LBW), and Odds for Small for Gestational Age (SGA) in Massachusetts from 2001 to 2013 (n = 629,675). Estimated effects and 95% confidence intervals are presented separately for those born to black mothers and those born to white mothers. Model covariates include: particulate matter under 2.5 µm in aerodynamic diameter ($PM_{2.5}$), clinical gestational age, mother's age, infant sex, year of birth, maternal marital status, Medicaid status, maternal smoking, gestational diabetes, other diabetes, high blood pressure during pregnancy, chronic high blood pressure, parity, mode of delivery, Kessner index for adequacy of prenatal care, and maternal education.

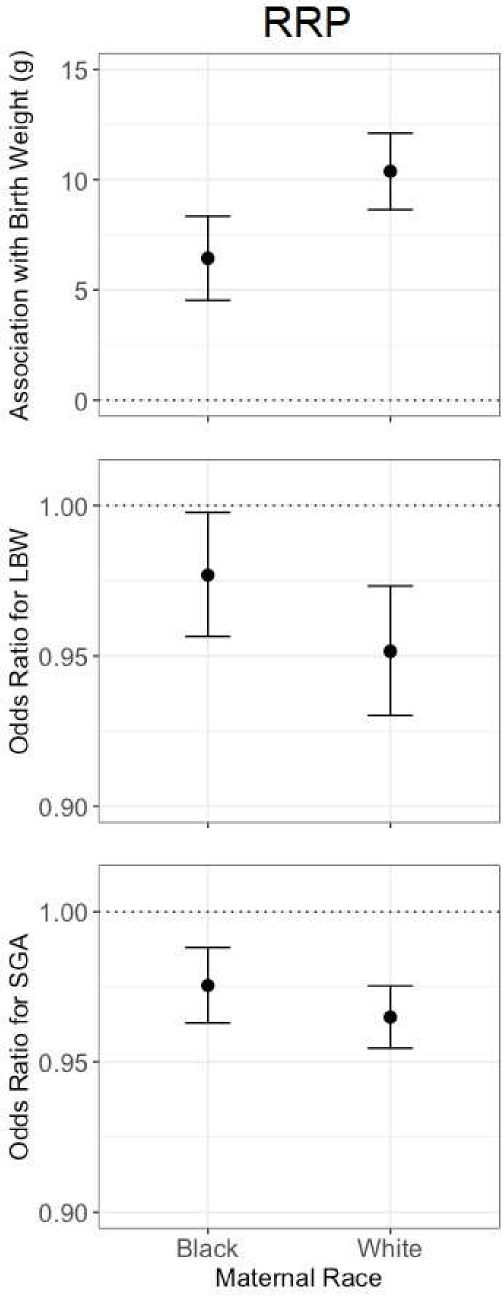

Figure S3.
Association between an Interquartile Range Increase in Census Block Group Economic Residential Polarization (RRP; 0.43) and Continuous Birth Weight, Odds for Low Birth Weight (LBW), and Odds for Small for Gestational Age (SGA) in Massachusetts from 2001 to 2013 (n = 629,675). Estimated effects and 95% confidence intervals are presented separately for those born to black mothers and those born to white mothers. Model covariates include: particulate matter under 2.5 μm in aerodynamic diameter ($PM_{2.5}$), clinical gestational age, mother's age, infant sex, year of birth, maternal marital status, Medicaid status, maternal smoking, gestational diabetes, other diabetes, high blood pressure during pregnancy, chronic high blood pressure, parity, mode of delivery, Kessner index for adequacy of prenatal care, and maternal education.

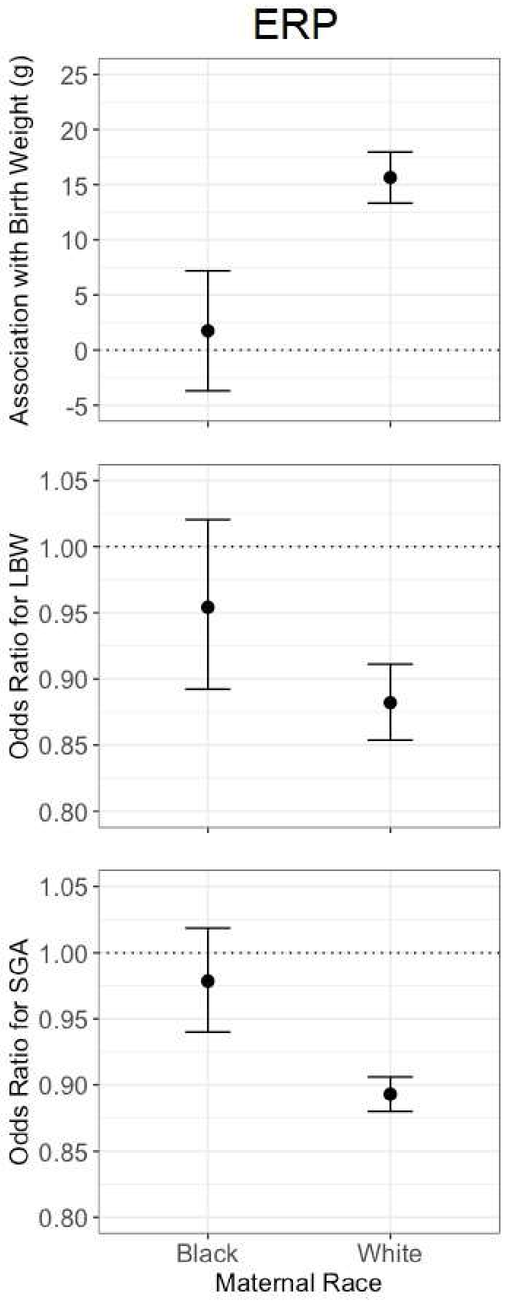

Supplement: Supplementary file 1 [file ijerph-17-03076-s001.pdf]
